# Analysis of influencing factors of indebtedness in Chinese private hospitals

**DOI:** 10.1097/MD.0000000000049434

**Published:** 2026-06-19

**Authors:** Fanna Kong, Jian Xu, Ke Ma

**Affiliations:** a School of Business, Jiangsu Ocean University, Lianyungang, Jiangsu, China; b International Business School, Qingdao Huanghai University, Qingdao, Shandong, China; c School of Economics and Management, Qingdao Agricultural University, Qingdao, Shandong, China; d College of Arts, Qingdao University of Science & Technology, Qingdao, Shandong, China.

**Keywords:** Chinese private hospital, debt level, influencing factor

## Abstract

This study aims to analyze how the indebtedness of Chinese private hospitals is influenced by hospital-specific variables, corporate governance variables, and macroeconomic variables. This study collects panel data from 13 Chinese private hospitals listed on the Shanghai and Shenzhen stock exchanges from 2012 to 2023 and uses the first difference fixed effects models. The level of indebtedness is measured through total, short-term, and long-term debt. Statistical significance is assessed using a threshold of *P* < .05 (2-tailed) for all analyses. The results show that employee expenses have a negative impact on short-term debt and a positive impact on long-term debt. Current ratio and growth rate of gross domestic product negatively influence total and short-term debt. In addition, no statistically significant impact is found between hospital size and corporate governance and the indebtedness of Chinese private hospitals. This study may enhance hospital managers’ awareness of debt levels while providing evidence-based strategies to optimize capital structure decisions.

## 1. Introduction

In recent years, the healthcare sector in China has undergone significant transformations, particularly with the development of private hospitals.^[1,2]^ These institutions play a crucial role in complementing the public healthcare system, meeting the diverse needs of the population, and driving innovation in medical services.^[3,4]^ In addition, China’s healthcare finance has also experienced profound transformations since 2012, driven by national health reforms under the Healthy China 2030 initiative. Chinese public hospitals dominate service provision and operate within a dual financing system combining government subsidies and market-oriented funding.^[5]^ However, the financing mechanisms of private hospitals differ fundamentally from public counterparts.^[6]^ Public hospitals enjoy government subsidies, tax exemptions, and preferential access to low-interest loans: fiscal advantages rooted in their public service mandate.^[7]^ These benefits are inherently nontransferable to private institutions. Private institutions face 40% higher financing costs than public counterparts due to limited collateral and credit ratings.^[8]^ In addition, for private hospitals, the bond market remains underutilized.^[9]^ Recent policy shifts, including expanded social welfare expenditure tax deductions and pilot public-private partnership programs in Shanghai and Guangdong, aim to alleviate financing constraints of private hospitals. However, systemic barriers persist, with most private hospitals relying on short-term bank loans despite long-term investment needs in medical equipment and talent retention. The financial health of private hospitals is often under scrutiny, with indebtedness emerging as a critical concern.^[10]^ High debt levels can jeopardize the operational stability and long-term sustainability of these hospitals, ultimately impacting patient care and healthcare access.^[11,12]^ Thus, understanding the factors influencing debt levels of Chinese private hospitals is of great importance for hospital managers, policymakers, and stakeholders.

The research on corporate capital structure originated from the theorem proposed by Modigliani and Miller,^[13]^ which posits that a company’s value remains unaffected by its financing method. Subsequently, Myers and Majluf^[14]^ proposed the pecking order theory, which suggests that firms prioritize financing sources based on information asymmetry and signaling costs. Companies prefer internal financing first, then debt, and view equity issuance as a last resort. This theory contrasts with Modigliani–Miller capital structure irrelevance by emphasizing real-world frictions. It remains influential in explaining corporate financing behavior, particularly for firms with limited transparency or volatile earnings. Based on this theory, this topic has been continuously analyzed in specific sectors (healthcare, real estate, tourism, food, etc).^[12,15–17]^ In addition, existing literature on hospital finance has predominantly focused on public hospitals, with limited studies examining private hospitals, especially in the Chinese context.^[12,18]^ Some studies have explored the relationship between size, financial performance, and indebtedness.^[12,15,17,19]^ However, these studies failed to comprehensively analyze the multifaceted factors that contribute to indebtedness.

To address these gaps, based on the pecking order theory, this study aims to provide a comprehensive analysis of the influencing factors of debt levels in Chinese private hospitals during the period from 2012 to 2023. These factors focus on 3 dimensions, including hospital-specific characteristics, corporate governance characteristics, and macroeconomic characteristics. Hospital-specific characteristics include employee expenses (EE), hospital size (SIZE), and current ratio (CR). Corporate governance characteristics include the existence of a Big Four auditing company (BIG) and board size (BS). Macroeconomic characteristics consist of the growth rate of gross domestic product (GDP) (GRG) and the consumer price index (CPI) to account for the broader economic environment’s impact on hospital financing. The GRG serves as a core indicator for assessing overall economic vitality. Economic growth will lead to an increase in long-term debt.^[20]^ The CPI acts as an inflation barometer. An increase in the inflation level will lead to an increase in corporate risks, which makes companies limit long-term debt.^[20]^ In analyzing these variables, we employ the first difference fixed effects models to investigate their relationships with total, short-term, and long-term debt. By doing so, we aim to provide a comprehensive picture of the financial challenges faced by Chinese private hospitals.

The contributions of this study are presented in 3 ways. Firstly, it fills a gap in the literature by focusing on private hospitals in China, a context that has received scant attention despite its growing importance. Secondly, by examining a range of influencing factors, including hospital-specific characteristics, corporate governance characteristics, and macroeconomic characteristics, we provide a more nuanced understanding of debt levels in private hospitals. Thirdly, our findings can offer practical implications for hospital managers and policymakers, suggesting strategies for debt management and risk mitigation. In terms of practical significance, our study can inform hospital managers about the financial implications of various strategies, such as reasonably controlling EE. Policymakers may use the findings to develop targeted support measures for private hospitals, enhancing their financial resilience and operational stability. Moreover, our research underscores the need for alternative financing channels and improved liquidity management, which can be critical in mitigating debt risks.

## 2. Methods

### 2.1. Sample and data

The sample in this study consists of Chinese private hospitals listed on the A-share market from 2012 to 2023. We excluded hospitals without relevant information on all variables, delisted hospitals, hospitals with a debt ratio > 1, and special treatment (ST) hospitals. In the Chinese stock market, ST companies typically refer to financially abnormal companies. When listed companies encounter financial issues such as consecutive losses or negative net assets, they are labeled as ST companies. These companies are subject to stricter regulatory oversight and face delisting risks. Finally, the sample consists of 13 Chinese private hospitals with 127 observations. The financial data are obtained from the CSMAR database, and macroeconomic variable information is collected from the World Bank. Our unbalanced panel data are analyzed using Stata 17.0. In this study, because human subject data are not involved, Institutional Review Board approval is not required.

### 2.2. Variable definition

Guided by Neves and Carolina,^[12]^ Kuc and Kalicanin,^[21]^ and Xu et al,^[22]^ indebtedness is measured by total liabilities (TL), short-term liabilities (STL), and long-term liabilities (LTL).

Regarding the influencing factors, hospital-specific variables comprise EE, SIZE, and CR, consistent with Neves and Carolina^[12]^ and Cheng et al.^[23]^ Few studies have investigated the relationship between EE and debt levels.^[12]^ These expenses are essential expenses that enterprises must bear during their operations and play a crucial role in the stable development of the company and employee motivation. They enable businesses to evaluate whether their human resource investments are reasonable while providing valuable references for financial management decisions.^[24]^ Some researchers argued that companies with strong social responsibility tend to have easier access to external financing.^[25,26]^ SIZE reflects institutional capacity and scale.^[7,21,27]^ CR serves as an indicator of liquidity and the hospital’s ability to meet short-term obligations.^[21,27–30]^ Guided by Neves and Carolina,^[12]^ corporate governance variables include BIG and BS. The Big Four auditing companies are generally regarded as benchmarks for audit quality. Their involvement can significantly reduce information asymmetry and enhance creditors’ trust in corporate financial transparency.^[31]^ Evidence suggests that companies audited by the Big Four auditing companies are more likely to obtain long-term debt.^[32]^ BS serves as a critical indicator of corporate governance efficiency. Empirical studies show a positive correlation between BS and debt levels.^[33]^ Macroeconomic variables consist of the GRG and the CPI. Table 1 shows the definition of all variables.

**Table 1:** Variable definition.

Variable	Symbol	Measurement
Total liabilities	TL	Total liabilities/total assets
Short-term liabilities	STL	Current liabilities/total assets
Long-term liabilities	LTL	Long-term liabilities/total assets
Employee expenses	EE	Employee expenses including wages, bonuses, allowances and subsidies, pension insurance, unemployment insurance, housing provident fund, housing benefit, and expenses paid for retired personnel/sales
Hospital size	SIZE	Natural logarithm of total assets
Current ratio	CR	Current assets/current liabilities
The existence of a Big Four auditing company	BIG	Dummy variable that takes 1 if a hospital is audited by a Big Four auditing company and 0 otherwise
Board size	BS	Number of board of directors
Growth rate of gross domestic product	GRG	(Current year’s gross domestic product-last year’s gross domestic product)/last year’s gross domestic product
Consumer price index	CPI	Consumer price index in each year

BIG = the existence of a Big Four auditing company; BS = board size; CPI = consumer price index; CR = current ratio; EE = employee expenses; GRG = growth rate of gross domestic product; LTL = long-term liabilities; SIZE = hospital size; STL = short-term liabilities; TL = total liabilities.

### 2.3. Models

Models (1)–(3) are used in this study. In Model (1), the dependent variable is TL. In Model (2), STL is the dependent variable, and LTL is the dependent variable in Model (3). Table 2 shows the model specification. By employing firm and year fixed effects, we control for unobserved heterogeneity and time-varying factors, ensuring the robustness of our findings. The augmented Dickey–Fuller test results indicate that the time series is stationary, and heteroskedasticity-robust standard errors are used in this study.

**Table 2:** Model specification.

No.	Model
1	TL = *β*_0_EE + *β*_1_SIZE + *β*_2_CR + *β*_3_BIG + *β*_4_BS + *β*_5_GRG + *β*_6_CPI + ∑FIRM + ∑YEAR + ε
2	STL = *β*_0_EE + *β*_1_SIZE + *β*_2_CR + *β*_3_BIG + *β*_4_BS + *β*_5_GRG + *β*_6_CPI + ∑FIRM + ∑YEAR + ε
3	LTL = *β*_0_EE + *β*_1_SIZE + *β*_2_CR + *β*_3_BIG + *β*_4_BS + *β*_5_GRG + *β*_6_CPI + ∑FIRM + ∑YEAR + ε

BIG = the existence of a Big Four auditing company, BS = board size, CPI = consumer price index, CR = current ratio, EE = employee expenses, FRIM = firm fixed effects, GRG = growth rate of gross domestic product, LTL = long-term liabilities, SIZE = hospital size, STL = short-term liabilities, TL = total liabilities, YEAR = year fixed effects.

## 3. Results

### 3.1. Descriptive statistics

Table 3 presents the descriptive statistics of all variables. TL has a mean value of 0.4285. STL has a mean value of 0.3235, and LTL has a mean value of 0.0929, which suggests that Chinese private hospitals are more dependent on short-term debt. The mean value of EE is 0.2584 with a maximum value of 0.5457 and a minimum value of 0.0467. Specifically, EE of Chinese private hospitals range from 20 million to 5 billion. CR has a mean value of 2.0752 with a high standard deviation, which suggests that the liquidity of Chinese private hospitals varies greatly. It is noted that most private hospitals are not audited by the Big Four auditing companies. BS has a mean value of 8.4094.

**Table 3:** Descriptive statistics.

Variable	Mean	Maximum	Minimum	SD
TL	0.4285	0.9901	0.0332	0.2283
STL	0.3235	0.9234	0.0304	0.2129
LTL	0.0929	0.3358	0	0.0886
EE	0.2584	0.5457	0.0467	0.1225
SIZE	21.8873	24.1307	20.0435	0.9128
CR	2.0752	27.5103	0.2729	3.0718
BIG	0.0236	1	0	0.1525
BS	8.4094	14	5	1.8574
GRG	0.0619	0.084	0.022	0.0188
CPI	0.0185	0.029	0.002	0.0078

BIG = the existence of a Big Four auditing company; BS = board size; CPI = consumer price index; CR = current ratio; EE = employee expenses; GRG = growth rate of gross domestic product; LTL = long-term liabilities; SD = standard deviation, SIZE = hospital size; STL = short-term liabilities; TL = total liabilities.

Table 4 presents the trend analysis of TL, STL, and LLT by year. In general, 3 variables showed an upward trend during the observed period. From 2012 to 2014, TL and STL showed a rising trend. Because of China’s supply-side structural reform in 2015, there was a temporary decline in TL and STL, while TL and STL continued to increase after 2017. The 3 variables reached their highest point during coronavirus disease 2019, which is consistent with the findings of Wang and Xu.^[10]^ The upward trend in these 3 variables requires managers’ attention.

**Table 4:** Descriptive statistics of TL, STL, and LLT by year.

Year	TL	STL	LLT
2012	0.3524	0.3122	0.0280
2013	0.3812	0.3221	0.0491
2014	0.4095	0.3627	0.0375
2015	0.3546	0.2919	0.0557
2016	0.3268	0.2700	0.0491
2017	0.3937	0.2945	0.0795
2018	0.4033	0.2624	0.1192
2019	0.4617	0.3316	0.1167
2020	0.4954	0.3732	0.1064
2021	0.5243	0.3612	0.1502
2022	0.4907	0.3430	0.1388
2023	0.4990	0.3420	0.1481

LTL = long-term liabilities; STL = short-term liabilities; TL = Total liabilities.

### 3.2. Correlation analysis

Table 5 describes the results of correlation analysis. We use Pearson correlation coefficients to assess the linear relationships between variables. EE is negatively correlated with TL and STL, while it is positively correlated with LTL. SIZE is positively correlated with TL and LTL. CR is negatively correlated with TL, STL, and LTL. BIG and BS are not correlated with indebtedness. In addition, all values of variance inflation factor are computed, and all of them are < 5, suggesting that there is no serious multicollinearity.

**Table 5:** Correlation matrix.

Variable	TL	STL	LTL	EE	SIZE	CR	BIG	BS	GRG	CPI	VIF
TL	1										-
STL	0.922*** (0.000)	1									-
LTL	0.347*** (0.000)	−0.034 (0.707)	1								-
EE	−0.196** (0.027)	−0.378*** (0.000)	0.380*** (0.000)	1							1.32
SIZE	0.238*** (0.007)	0.124 (0.167)	0.306*** (0.000)	0.276*** (0.002)	1						1.35
CR	−0.496*** (0.000)	−0.438*** (0.000)	−0.202** (0.023)	0.048 (0.591)	−0.198** (0.026)	1					1.11
BIG	0.079 (0.379)	0.064 (0.474)	0.068 (0.445)	0.178** (0.046)	0.302*** (0.001)	−0.057 (0.528)	1				1.20
BS	0.112 (0.210)	0.133 (0.135)	0.021 (0.813)	0.108 (0.227)	0.118 (0.188)	0.183** (0.040)	0.218** (0.014)	1			1.12
GRG	−0.144 (0.107)	−0.059 (0.512)	−0.230*** (0.009)	−0.398*** (0.000)	−0.292*** (0.000)	0.054 (0.550)	−0.054 (0.543)	−0.043 (0.628)	1		1.31
CPI	−0.106 (0.237)	−0.019 (0.831)	−0.237*** (0.007)	−0.162* (0.068)	−0.209** (0.018)	0.079 (0.377)	0.050 (0.581)	−0.084 (0.349)	−0.068 (0.447)	1	1.14

BIG = the existence of a Big Four auditing company, BS = board size, CPI = consumer price index, CR = current ratio, EE = employee expenses, GRG = growth rate of gross domestic product, LTL = long-term liabilities, SIZE = hospital size, STL = short-term liabilities, TL = total liabilities, VIF = variance inflation factor.

* *P* < .10.

** *P* < .05.

*** *P* < .01. *P* values are in parentheses.

### 3.3. Regression results

Table 6 shows the regression results of Model (1). Although the coefficient of EE is positive (*β* = 0.031, *P* = .869), it does not reach the significance level of 5%, suggesting that EE do not increase the overall debt level. Regarding SIZE, there is a negative but nonsignificant relationship between SIZE and TL (*β* = −0.017, *P* = .517). CR has a negative relationship with total indebtedness (*β* = −0.019, *P* = .012). Corporate governance variables have no significant impact on TL. GRG shows a negative and significant relationship with TL (*β* = −2.733, *P* = .049).

**Table 6:** Regression results of Model (1).

Variable	Coefficient	Robust SE	*t*	*P*
Constant	1.138*	0.605	1.88	.063
EE	0.031	0.189	0.17	.869
SIZE	−0.017	0.026	−0.65	.517
CR	−0.019**	0.007	−2.57	.012
BIG	0.044	0.060	0.74	.462
BS	0.003	0.013	0.24	.810
GRG	−2.733**	1.368	−2.00	.049
CPI	−3.476	2.323	−1.50	.138
Firm fixed effects	Yes			
Year fixed effects	Yes			
*R* ^2^	0.7203			
Adjusted *R*^2^	0.6404			
*F*	52.86***			
Joint significance test	2.26**			

BIG = the existence of a Big Four auditing company, BS = board size, CPI = consumer price index, CR = current ratio, EE = employee expenses, GRG = growth rate of gross domestic product, SE = standard error, SIZE = hospital size.

* *P* < .10.

** *P* < .05.

*** *P* < .01.

Table 7 presents the regression results of Model (2) with STL being the dependent variable. EE, CR, and GRG negatively influence STL, while the impact of corporate governance variables is not statistically significant.

**Table 7:** Regression results of Model (2).

Variable	Coefficient	Robust SE	*t*	*P*
Constant	1.491**	0.599	2.49	.014
EE	−0.285*	0.148	−1.93	.056
SIZE	−0.038	0.025	−2.53	.128
CR	−0.016***	0.006	−2.70	.008
BIG	0.070	0.057	1.21	.229
BS	0.008	0.014	0.60	.547
GRG	−2.792**	1.198	−2.33	.022
CPI	−2.547	2.070	−1.23	.221
Firm fixed effects	Yes			
Year fixed effects	Yes			
*R* ^2^	0.7541			
Adjusted *R*^2^	0.6838			
*F*	42.30***			
Joint significance test	2.39**			

BIG = the existence of a Big Four auditing company, BS = board size, CPI = consumer price index, CR = current ratio, EE = employee expenses, GRG = growth rate of gross domestic product, SE = standard error, SIZE = hospital size.

* *P* < .10.

** *P* < .05.

*** *P* < .01.

Table 8 shows the regression results of Model (3). The adjusted *R*^2^ value in Model (3) is lower than that in Models (1) and (2). Model (3) focuses on long-term debt, which is inherently more stable and influenced by strategic decisions (e.g., long-term financing plans) rather than short-term operational factors. Factors such as hospitals’ long-term investment strategies or policy changes are not included in the model due to data limitations. EE has a positive impact on TLT (*β* = 0.245, *P* = .044). The coefficient of SIZE is 0.019, with a *P* value of .106. While this *P* value exceeds the conventional threshold of 0.05, it indicates a weak positive association between SIZE and long-term debt. This insignificance could be because of our small sample size. In addition, corporate governance variables and macroeconomic variables have no significant impact on LTL.

**Table 8:** Regression results of Model (3).

Variable	Coefficient	Robust SE	*t*	*P*
Constant	−0.284	0.276	−1.03	.306
EE	0.245**	0.120	2.04	.044
SIZE	0.019	0.011	1.63	.106
CR	−0.002	0.002	−1.13	.260
BIG	−0.015	0.025	−0.62	.539
BS	−0.001	0.005	−0.22	.826
GRG	−0.256	0.714	−0.36	.720
CPI	−1.258	1.209	−1.04	.300
Firm fixed effects	Yes			
Year fixed effects	Yes			
*R* ^2^	0.5957			
Adjusted *R*^2^	0.4802			
*F*	103.21***			
Joint significance test	3.85***			

BIG = the existence of a Big Four auditing company, BS = board size, CPI = consumer price index, CR = current ratio, EE = employee expenses, GRG = growth rate of gross domestic product, SE = standard error, SIZE = hospital size.

* *P* < .10.

** *P* < .05.

*** *P* < .01.

### 3.4. Robustness check

We use random effects models to reestimate all models, and the results are consistent with those shown in Tables 6–8, which suggests the robustness of our results.

## 4. Discussion

### 4.1. Result discussion

Firstly, EE exert no significant impact on total debt, while they demonstrate opposing effects on short-term and long-term debt. Specifically, there is a negative relationship between EE and short-term debt. Private hospitals rely heavily on medical technical teams and service quality as their core competitiveness, making employee salaries, training, and welfare a substantial portion of operating costs. When private hospitals increase EE, this often coincides with enhanced service capacity and patient satisfaction, thereby boosting revenue generation. This incremental revenue can be prioritized to repay STL, reducing reliance on short-term financing. However, EE have a positive impact on long-term debt. To attract and retain high-quality medical talent, private hospitals must offer more competitive long-term incentives (e.g., equity incentives, career development plans), necessitating long-term financial planning. Meanwhile, workforce stability and expansion are often aligned with strategic goals, such as establishing new departments, introducing advanced equipment, or expanding facilities, which requires long-term loans. Thus, increased EE may signal hospital expansion, prompting financial institutions to provide greater long-term financing support. The insignificant impact of EE on total debt can be explained by the offset between the reduction in short-term debt and the increase in long-term debt. Conversely, Cheng et al^[23]^ argued that social spending increases the need for additional funding.

Secondly, the insignificant impact of SIZE on their indebtedness stems from their unique operational characteristics and capital operation logic. Private hospitals’ financing channels are highly market-oriented, with their debt scale more dependent on shareholders’ investment and flexibility of private lending rather than solely relying on bank credit. It is reported that over 40% of private hospitals’ financing structure comes from equity financing, significantly higher than public hospitals.^[34]^ This capital structure enables hospital expansion more through internal accumulation rather than debt leverage. In addition, private hospitals generally adopt an “asset-light” operational model, reducing fixed asset investments through equipment leasing and outsourcing noncore services.^[35]^ This model allows hospitals to expand bed capacity or establish new departments without significantly increasing long-term debt. Meanwhile, their short-term debt primarily manifests as accounts payable and commercial credit financing, which naturally aligns with revenue scale. Revenue growth from scale expansion can offset short-term debt pressures. However, using a sample of Portuguese hospitals, Neves and Carolina^[12]^ found a negative relationship between size and total debt and a positive relationship between size and short-term and long-term debt.

Thirdly, there is a significantly negative relationship between CR and total and short-term debt. Pecking order theory suggests that companies with higher liquidity prefer to use internal financing, which leads to less dependence on external capital. Liquidity is a crucial variable for Chinese private hospitals to maintain financial stability.^[36]^ This is consistent with Neves and Carolina^[12]^ and Khaki and Akin.^[37]^

Fourthly, corporate governance variables have no significant impact on the level of hospital debt. The insignificance of BIG might be caused by the fact that few hospitals are audited by large and well-known audit firms. Huq et al^[38]^ also pointed out that such auditing firms do not bring any additional cost to the benefits of debt. Regarding BS, there is also no significant relationship with hospital indebtedness. However, Neves and Carolina^[12]^ found a positive relationship between the size of the board of directors and STL. Kang and Ausloos,^[39]^ taking Chinese firms as the sample, found that short-term and long-term debt are negatively related to BS.

Finally, the GRG has a significant and negative impact on total and short-term debt, suggesting that private hospitals adopt countercyclical financing strategies during economic expansions. From the perspective of short-term debt, when GDP growth is high, increased economic activity enhances residents’ ability to afford medical services, driving up outpatient volumes and demand for high-value medical procedures in private hospitals. This improves operational cash flow, enabling hospitals to cover liquidity expenses such as pharmaceutical procurement and staff salaries through internal revenue, thereby reducing reliance on short-term financing. Regarding TL, the reduction in STL directly lowers overall debt levels, while the stability of LTL acts as a counterbalance. Regarding the insignificant impact on long-term debt, long-term debt primarily finances fixed asset investments. Expansion plans for private hospitals typically hinge on noncyclical factors such as regional healthcare demand forecasts, licensing approval timelines, and technology upgrade cycles for equipment: all of which have limited correlation with short-term GDP fluctuations. Furthermore, long-term loans involve lengthy approval processes and stringent conditions, with lenders prioritizing structural indicators like institutional qualifications and cash flow stability over transient economic growth rates.

### 4.2. Practical implications

The findings of this study have some policy implications for Chinese private hospitals. Firstly, it is crucial for these hospitals to practice meticulous financial management, particularly in managing EE. Optimizing staffing costs through performance-based incentives or skill-mix adjustments could reduce debt dependency while maintaining service quality. Additionally, aligning debt maturities with operational cash flow cycles (such as using long-term loans for equipment procurement and short-term financing for working capital) may enhance financial resilience.

Secondly, enhancing liquidity is another vital strategy to mitigate debt risks. Chinese private hospitals can explore diverse financing channels, such as collaborating with financial institutions or seeking investments from private equity firms, to improve their liquidity position. Additionally, establishing good relationships with Big Four auditing companies may bring certain benefits, although the impact is not statistically significant in our study.

Finally, the government and relevant regulatory authorities could play a role in facilitating the financing of private hospitals by providing more policy support and creating a conducive financing environment. Overall, a comprehensive approach combining careful financial management, strategic financing, and policy support is essential for the sustainable development of Chinese private hospitals. In addition, policymakers should recognize the counterintuitive negative link between GDP growth and short-term debt. During economic upturns, targeted interventions like low-interest long-term loans or tax breaks for capital investments could incentivize hospitals to shift from short-term to stable funding sources. Simultaneously, strengthening social welfare programs (e.g., public medical insurance integration) may reduce patient payment delays, directly improving hospital liquidity.

### 4.3. Limitations and future research

This study has its limitations. The sample size is relatively small, consisting of only 13 hospitals, which may limit the generalizability of our findings. Future research could expand the sample to unlisted hospitals and conduct comparative analysis with Vietnamese/Indian private hospitals operating under similar dual-track healthcare systems. Additionally, while we consider various influencing factors, other potential variables, such as service performance and hospital management strategies,^[40]^ may also play significant roles in debt levels. Future research could incorporate more diverse factors to deepen our comprehension of this complex issue.

## 5. Conclusions

This study aims to analyze the influencing factors of indebtedness in Chinese private hospitals, drawing upon panel data from 13 private hospitals listed on the Shanghai and Shenzhen stock exchanges spanning from 2012 to 2023. By employing the first difference fixed effects models, we examine the impact of hospital-specific variables, corporate governance variables, and macroeconomic variables on the debt levels of these hospitals, measured through total, short-term, and LTL. Our key findings reveal several notable insights. Firstly, EE negatively influence short-term debt and positively affect long-term debt. Secondly, CR demonstrates a significant negative relationship with indebtedness. Thirdly, GRG has a negative impact on total and short-term debt. Finally, SIZE and corporate governance variables do not significantly impact hospital debt levels. This study will help hospital managers improve capital structure and profitability.

## Author contributions

**Conceptualization:** Fanna Kong, Jian Xu.

**Data curation:** Jian Xu.

**Formal analysis:** Jian Xu.

**Funding acquisition:** Fanna Kong.

**Project administration:** Fanna Kong.

**Resources:** Fanna Kong.

**Software:** Jian Xu.

**Supervision:** Jian Xu.

**Validation:** Jian Xu.

**Visualization:** Jian Xu.

**Writing** – **original draft:** Fanna Kong, Jian Xu, Ke Ma.

**Writing** – **review & editing:** Jian Xu.
